# 2356 The nasopharyngeal microbiome is perturbed and associated with increased clinical severity during acute respiratory viral infection

**DOI:** 10.1017/cts.2018.137

**Published:** 2018-11-21

**Authors:** Darrell Dinwiddie, Ashlee K. Bradley, Jesse L. Denson, Joshua L. Kennedy, Walter N. Dehority, Kurt C. Schwalm, Stephen A. Young

**Affiliations:** Clinical and Translational Science Center, University of New Mexico

## Abstract

OBJECTIVES/SPECIFIC AIMS: We sought to investigate the role of the host microbiome during severe, acute respiratory infection (ARI) to understand the drivers of both acute clinical pathogenesis. METHODS/STUDY POPULATION: Nasopharyngeal swabs comprised of mixed cell populations at the active site of infection were collected from 192 hospitalized pediatric patients with ARI. We combined comprehensive respiratory virus detection and virus genome sequencing with 16S rRNA gene sequencing to evaluate the microbial content of the airway during ARI. This data was coupled with 11 clinical parameters, which were compiled to create a clinical severity score. The microbiome profiles were assessed to determine if clinical severity of infection, and/or specific virus was associated with increased clinical severity. RESULTS/ANTICIPATED RESULTS: We identified 8 major microbiome profiles classified by dominant bacterial genus, Moraxella, Corynebacterium, Staphylococcus, Haemophilus, Streptococcus, Alloiococcus, Schlegelella, and Diverse. Increased clinical severity was significantly associated with microbiome profiles dominated by Haemophilus, Streptococcus, and Schlegelella, whereas Corynebacterium and Alloiococcus were more prevalent in children with less severe disease. Independent of the microbial community, more than 60% of patients with the highest clinical severity were infected with either respiratory syncytial virus or rhinovirus. DISCUSSION/SIGNIFICANCE OF IMPACT: Our results indicate that individually and in combination, both virus and microbial composition may drive clinical severity during acute respiratory viral infections. It is still unclear how the complex interplay between virus, bacterial community, and the host response influence long-term respiratory impacts, such as the development of asthma. Nonetheless, during ARIs therapeutic interventions such as antibiotics and probiotics may be warranted in a subset of patients that are identified to have both a virus and microbiome profile that is associated with increased pathogenesis to limit both acute and long-term phenotypes.

